# Neoadjuvant chemoimmunotherapy in resectable locally advanced oral squamous cell carcinoma

**DOI:** 10.1097/JS9.0000000000002826

**Published:** 2025-06-30

**Authors:** Wang Shuhan, Dong Gang, Ma Xiangrui

**Affiliations:** a School of Stomatology,Qilu Medical University, ZiBo, Shandong, PR China; b Department of Oral and Maxillofacial Surgery, Binzhou Medical University Hospital, Binzhou, Shandong, PR China

We read with great interest the study by Li *et al* (2024), titled *“Neoadjuvant chemoimmunotherapy in resectable locally advanced oral squamous cell carcinoma: a single-center retrospective cohort study”*^[1]^. This study investigates the impact of neoadjuvant chemoimmunotherapy (NACI) on the treatment of locally advanced resectable oral squamous cell carcinoma (OSCC). By combining immune checkpoint inhibitors (ICIs) with conventional chemotherapy, the authors demonstrate significant improvements in major pathological response (MPR), pathological complete response (PCR), and overall patient survival rates. Li *et al* provide compelling evidence supporting the efficacy of NACI in enhancing oncological outcomes and paving the way for more personalized treatment strategies^[2]^

The efficacy of NACI is highlighted by its ability to achieve a PCR rate of 47.1% and an MPR rate of 65.4%, surpassing historical benchmarks for neoadjuvant chemotherapy alone. Traditional chemotherapy approaches have reported MPR rates between 30% and 40%^[3,4]^. Furthermore, the observed improvements in disease-free survival (DFS) (89.0% vs. 60.8%) and overall survival (OS) (91.3% vs. 64.0%) underscore the potential of NACI to redefine preoperative treatment standards. These findings align with previous systematic reviews and meta-analyses confirming that chemoimmunotherapy enhances PCR and partial response rates compared to chemotherapy alone. This growing body of evidence further supports the incorporation of novel therapeutic combinations that can boost immunogenicity and improve long-term patient outcomes.

Beyond clinical efficacy, the study also explores predictive biomarkers for treatment response. The authors identify the PD-L1 combined positive score (CPS) and total cholesterol levels as significant indicators of therapeutic efficacy. Higher CPS values have consistently correlated with improved pathological responses, supporting their potential utility as biomarkers for patient stratification^[5,6]^. Additionally, the observed inverse correlation between cholesterol levels and treatment efficacy suggests a potential metabolic component influencing immune modulation. These findings highlight the need to further explore metabolic and molecular biomarkers to refine patient selection for NACI. Identifying additional predictive biomarkers, such as tumor mutational burden (TMB) and immune cell infiltration profiles, could provide a more comprehensive understanding of treatment response dynamics and offer insights into mechanisms of therapy resistance^[7]^.

The safety profile of NACI is another critical consideration. The study reports that most treatment-related adverse events (TRAEs) were mild (Grade 1-2), including alopecia (100%), anemia (77.9%), and pruritus (59.6%). Only one patient experienced Grade 3-4 TRAEs, and no treatment-related fatalities were observed^[1]^. These findings reinforce those of previous systematic reviews, which indicate that NACI does not significantly delay surgical interventions or increase the risk of severe adverse effects. Nevertheless, immune-related adverse events (irAEs), such as endocrinopathies and pneumonitis, warrant further investigation to ensure long-term patient safety and well-being^[8,9]^. As ICIs become more widely adopted in oncology, understanding and managing long-term toxicities will be critical to their broader clinical integration. Additionally, exploring alternative dosing schedules and combination regimens may offer opportunities to optimize efficacy while minimizing toxicity.

While the findings are promising, the study’s single-center design may limit its generalizability. Larger, multicenter randomized controlled trials (RCTs) are needed to validate these results and confirm their applicability to diverse patient populations^[10,11]^. Moreover, the median follow-up duration of 31 months provides encouraging preliminary evidence, but longer observation is required to assess long-term recurrence patterns and survival outcomes. Recent phase II trials, including those by Zhang *et al* (2022) and Wang *et al* (2023), underscore the potential of NACI to improve survival trends and emphasize the necessity for continued research in this area^[10,12]^. Future studies should also investigate potential variations in NACI response based on genetic predispositions, tumor microenvironment heterogeneity, and different clinical subtypes of OSCC, all of which may influence treatment efficacy^[7]^.

Future research should focus on refining treatment protocols, optimizing combination therapies, and identifying additional predictive biomarkers. The use of artificial intelligence (AI) and machine learning to analyze large-scale patient data may further enhance treatment stratification and predictive accuracy^[13]^. AI-driven models have shown promise in predicting therapy responses based on multi-omic data, integrating genomic, transcriptomic, and proteomic information^[4]^. Moreover, understanding the role of the tumor microenvironment in modulating immune responses could lead to the development of more targeted therapeutic interventions, further enhancing treatment efficacy across patient subgroups^[14]^. Personalized treatment strategies leveraging AI-generated insights may significantly improve the precision of neoadjuvant therapeutic decision-making and minimize unnecessary toxicity.

Another area of focus should be the economic feasibility of NACI. Given the high cost of ICIs, it is crucial to assess the cost-effectiveness of NACI relative to conventional neoadjuvant strategies. Policymakers and healthcare providers must ensure equitable access to these therapies, particularly for patients in resource-limited settings^[1,12]^. Future studies should evaluate cost-benefit ratios and identify patient subgroups that derive the greatest benefit from NACI to optimize healthcare resource allocation. The integration of pharmacoeconomic modeling could provide valuable insights into the sustainability of widespread NACI implementation. Moreover, increasing accessibility through generic versions of key drugs or financial assistance programs may further improve patient affordability and treatment adherence.

Additionally, investigating immune resistance mechanisms and strategies to overcome them remains a priority^[14,15]^. The interplay between immune checkpoint blockade, TMB, and other emerging biomarkers could provide deeper insights into why certain patients respond more favorably to NACI. Exploring innovative combination regimens—such as incorporating cytokine modulators, metabolic inhibitors, or gut microbiota-targeting interventions—may further enhance treatment efficacy and reduce relapse rates^[15–17]^. Overcoming both primary and acquired resistance mechanisms remains a challenge, and future studies should focus on elucidating the pathways that contribute to resistance and investigating novel therapeutic combinations to enhance response durability. Understanding resistance at the molecular level could facilitate the development of personalized therapeutic strategies tailored to an individual’s tumor biology.

The impact of NACI on post-surgical recovery and long-term quality of life is another area requiring further study^[18,19]^. While the treatment has demonstrated improved survival rates, its influence on surgical outcomes, postoperative complications, and functional recovery must be thoroughly examined. Patients undergoing extensive surgical procedures following NACI may experience different healing responses compared to those receiving conventional treatments^[20]^. Understanding these dynamics is essential for optimizing preoperative planning and postoperative care, ultimately leading to better functional and aesthetic outcomes. Additional studies examining the interaction between advances in surgical techniques and the effects of neoadjuvant therapy will be crucial for optimizing patient outcomes, particularly in reconstructive surgery cases where tissue viability may be influenced by prior treatments^[20]^.

Ultimately, the evolving role of NACI in OSCC management necessitates a collaborative, multidisciplinary approach. Advances in molecular profiling, real-time monitoring of immune responses, and the development of novel immunotherapeutic agents hold the potential to reshape treatment paradigms. By integrating cutting-edge research across oncology, immunology, and computational biology, the oncology community can continue to refine NACI strategies to maximize patient benefits and achieve long-term success in clinical applications. With continued progress, the application of NACI may expand beyond OSCC to other solid tumors, further broadening its clinical impact and reinforcing its role in personalized cancer therapy.

## Data Availability

No new data were generated for this study.
